# 3,3′-(1,4-Phenyl­ene)bis­[2-(propyl­amino)­benzofuro[3,2-*d*]pyrimidin-4(3*H*)-one] ethanol disolvate

**DOI:** 10.1107/S160053681201375X

**Published:** 2012-04-04

**Authors:** Li Li, Yong-Nian Qu, Jian Gong, Yang-Gen Hu

**Affiliations:** a The Library of Hubei University of Medicine, Shiyan 442000, People’s Republic of China; b Institute of Medicinal Chemistry, Hubei University of Medicine, Shiyan 442000, People’s Republic of China; c Institute of Basic Medical Sciences, Hubei University of Medicine, Shiyan 442000, People’s Republic of China; d Department of Pharmacy, Taihe Hospital of Hubei University of Medicine, Shiyan 442000, People’s Republic of China

## Abstract

The title compound, C_32_H_28_N_6_O_4_·2C_2_H_5_OH, consists of two 2-(propyl­amino)­benzofuro[3,2-*d*]pyrimidin-4(3*H*)-one units connected, *via* one of the pyrimidine N atoms, to a bridging benzene ring in the 1,4 positions. Two ethanol solvent mol­ecules are also present. The main mol­ecule lies on a center of symmetry located at the center of the benzene ring. The fused-ring system of the benzofuro[3,2-*d*]pyrimidine moiety is nearly planar (r.m.s. deviation = 0.016 Å) and forms a dihedral angle of 78.21 (7)° with the central benzene ring. The crystal structure features O—H⋯O and N—H⋯O inter­actions. The C atoms of the propyl­amino side chain in the main mol­ecule and the ethyl group in the solvent mol­ecule are disordered over two positions, with site-occupancy factors 0.34:0.66 and 0.42:0.58, respectively.

## Related literature

The title compound may be used as a precursor for obtaining bioactive mol­ecules with antitumor activity, see: Bellarosa *et al.* (1996) ▶. For the biological activity of benzofuropyrimidine derivatives, see: Moneam *et al.* (2004 ▶); Bodke & Sangapure (2003 ▶). For the crystal structures of other fused pyrimidinone derivatives, see: Hu *et al.* (2005 ▶, 2006 ▶, 2007 ▶, 2008 ▶). 
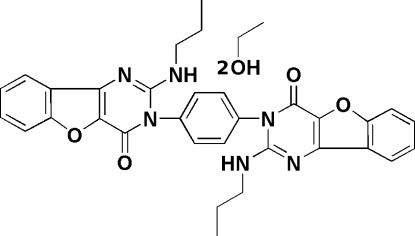


## Experimental

### 

#### Crystal data

C_32_H_28_N_6_O_4_·2C_2_H_6_O
*M* *_r_* = 652.74
Monoclinic, 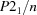

*a* = 10.1933 (12) Å
*b* = 13.6224 (16) Å
*c* = 12.5249 (15) Å
β = 105.409 (2)°
*V* = 1676.7 (3) Å^3^
*Z* = 2
Mo *K*α radiation
μ = 0.09 mm^−1^
*T* = 298 K
0.14 × 0.12 × 0.10 mm

#### Data collection

Bruker SMART 4K CCD area-detector diffractometer
Absorption correction: multi-scan (*SADABS*; Sheldrick, 2003 ▶) *T*_min_ = 0.988, *T*_max_ = 0.991
10883 measured reflections
2941 independent reflections
2327 reflections with *I* > 2σ(*I*)
*R*_int_ = 0.060

#### Refinement

*R*[*F* ^2^ > 2σ(*F* ^2^)] = 0.054
*wR*(*F* ^2^) = 0.151
*S* = 1.03
2941 reflections
259 parameters
60 restraints
H-atom parameters constrained
Δρ_max_ = 0.17 e Å^−3^
Δρ_min_ = −0.23 e Å^−3^

### 

Data collection: *SMART* (Bruker, 2001 ▶); cell refinement: *SAINT-Plus* (Bruker, 2001 ▶); data reduction: *SAINT-Plus*; program(s) used to solve structure: *SHELXS97* (Sheldrick, 2008 ▶); program(s) used to refine structure: *SHELXL97* (Sheldrick, 2008 ▶); molecular graphics: *PLATON* (Spek, 2009 ▶); software used to prepare material for publication: *SHELXTL* (Sheldrick, 2008 ▶).

## Supplementary Material

Crystal structure: contains datablock(s) I, global. DOI: 10.1107/S160053681201375X/lr2054sup1.cif

Structure factors: contains datablock(s) I. DOI: 10.1107/S160053681201375X/lr2054Isup2.hkl

Supplementary material file. DOI: 10.1107/S160053681201375X/lr2054Isup3.cml

Additional supplementary materials:  crystallographic information; 3D view; checkCIF report

## Figures and Tables

**Table 1:** Hydrogen-bond geometry (Å, °)

*D*—H⋯*A*	*D*—H	H⋯*A*	*D*⋯*A*	*D*—H⋯*A*
N3—H3a⋯O3	0.86	2.22	2.996 (3)	150
O3—H3b⋯O1^i^	0.82	2.12	2.903 (3)	159

Symmetry code: (i) 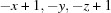.

**Fig. 1.**
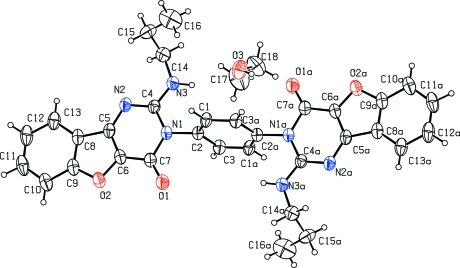
The molecular structure of the title compound. Displacement ellipsoids are drawn at the 50% probability level.

## supplementary crystallographic information

### Comment

As a part of our ongoing work in the preparation of derivatives of heterocyclic compounds (Hu *et al.*, 2005, 2006, 2007, 2008), we have synthesized and structurally characterized the title compound (Fig. 1). Here we wish to report an X-ray crystal structure of it (Fig. 1). In the molecule, the fused rings system of the benzo[4,5]furo[3,2-*d*]pyrimidine system are nearly coplanar (r.m.s. deviation= 0.016 A°), forming a dihedral angle of 78.21 (7)° with the (C1/C2/C3/C1a/C2a/C3a) phenyl ring . The crystal structure is stabilize by O—H···O and N—H···O hydrogen bonds. The C atoms of the propylamino side chain in molecule and the ethyl in solvent molecule are disordered over two positions, with site occupancy factors 0.34/0.66 and 0.42/58 for the C atoms of the propyl and the ethyl, respectively.

### Experimental

The compound was synthesized according to the procedures previously described in the literature (Hu *et al.*, 2005, 2006, 2007, 2008).

### Refinement

All H-atoms were positioned with idealized geometry and refined isotropic (*U*_iso_(H)= 1.5*U*_eq_(C)for methyl H atoms and *U*_iso_(H) =1.2*U*_eq_(C) for all other H atoms) using a riding model with C—H = 0.93°, 0.97° and 0.96 Å, O—H = 0.82°, N—H = 0.86°.

### Crystal data

**Table:**

C_32_H_28_N_6_O_4_·2C_2_H_6_O	*F*(000) = 692
*M**_r_* = 652.74	*D*_x_ = 1.293 Mg m^−^^3^
Monoclinic, *P*2_1_/*n*	Mo *K*α radiation, λ = 0.71073 Å
Hall symbol: -P 2yn	Cell parameters from 3984 reflections
*a* = 10.1933 (12) Å	θ = 2.3–26.6°
*b* = 13.6224 (16) Å	µ = 0.09 mm^−^^1^
*c* = 12.5249 (15) Å	*T* = 298 K
β = 105.409 (2)°	Block, colorless
*V* = 1676.7 (3) Å^3^	0.14 × 0.12 × 0.10 mm
*Z* = 2	

### Data collection

**Table:**

Bruker SMART 4K CCD area-detector diffractometer	2941 independent reflections
Radiation source: fine-focus sealed tube	2327 reflections with *I* > 2σ(*I*)
Graphite monochromator	*R*_int_ = 0.060
φ and ω scans	θ_max_ = 25.0°, θ_min_ = 2.3°
Absorption correction: multi-scan (*SADABS*; Sheldrick, 2003)	*h* = −12→12
*T*_min_ = 0.988, *T*_max_ = 0.991	*k* = −13→16
10883 measured reflections	*l* = −14→14

### Refinement

**Table:**

Refinement on *F*^2^	Primary atom site location: structure-invariant direct methods
Least-squares matrix: full	Secondary atom site location: difference Fourier map
*R*[*F*^2^ > 2σ(*F*^2^)] = 0.054	Hydrogen site location: inferred from neighbouring sites
*wR*(*F*^2^) = 0.151	H-atom parameters constrained
*S* = 1.03	*w* = 1/[σ^2^(*F*_o_^2^) + (0.0753*P*)^2^ + 0.3428*P*] where *P* = (*F*_o_^2^ + 2*F*_c_^2^)/3
2941 reflections	(Δ/σ)_max_ = 0.001
259 parameters	Δρ_max_ = 0.17 e Å^−^^3^
60 restraints	Δρ_min_ = −0.23 e Å^−^^3^

### Special details

**Table:**

Geometry. All e.s.d.'s (except the e.s.d. in the dihedral angle between two l.s. planes) are estimated using the full covariance matrix. The cell e.s.d.'s are taken into account individually in the estimation of e.s.d.'s in distances, angles and torsion angles; correlations between e.s.d.'s in cell parameters are only used when they are defined by crystal symmetry. An approximate (isotropic) treatment of cell e.s.d.'s is used for estimating e.s.d.'s involving l.s. planes.
Refinement. Refinement of *F*^2^ against ALL reflections. The weighted *R*-factor *wR* and goodness of fit *S* are based on *F*^2^, conventional *R*-factors *R* are based on *F*, with *F* set to zero for negative *F*^2^. The threshold expression of *F*^2^ > σ(*F*^2^) is used only for calculating *R*-factors(gt) *etc*. and is not relevant to the choice of reflections for refinement. *R*-factors based on *F*^2^ are statistically about twice as large as those based on *F*, and *R*- factors based on ALL data will be even larger.

### Fractional atomic coordinates and isotropic or equivalent isotropic displacement parameters (Å^2^)

**Table:**

	*x*	*y*	*z*	*U*_iso_*/*U*_eq_	Occ. (<1)
C1	0.6148 (2)	0.02496 (15)	0.58223 (16)	0.0567 (5)	
H1	0.6919	0.0422	0.6376	0.068*	
C2	0.53256 (19)	0.09654 (14)	0.52147 (15)	0.0506 (5)	
C3	0.4179 (2)	0.07207 (15)	0.43955 (16)	0.0561 (5)	
H3	0.3628	0.1210	0.3991	0.067*	
C4	0.6653 (2)	0.24409 (15)	0.50513 (16)	0.0538 (5)	
C5	0.6309 (2)	0.38471 (15)	0.58744 (16)	0.0546 (5)	
C6	0.5310 (2)	0.34514 (15)	0.62721 (17)	0.0594 (6)	
C7	0.4899 (2)	0.24663 (16)	0.60962 (17)	0.0588 (5)	
C8	0.6399 (2)	0.48678 (14)	0.62188 (17)	0.0587 (6)	
C9	0.5417 (3)	0.49804 (15)	0.67922 (18)	0.0654 (6)	
C10	0.5178 (3)	0.58535 (18)	0.7266 (2)	0.0794 (7)	
H10	0.4514	0.5909	0.7647	0.095*	
C11	0.5975 (3)	0.66348 (18)	0.7143 (2)	0.0870 (9)	
H11	0.5855	0.7236	0.7456	0.104*	
C12	0.6954 (3)	0.65482 (17)	0.6563 (2)	0.0861 (9)	
H12	0.7473	0.7094	0.6494	0.103*	
C13	0.7177 (3)	0.56759 (16)	0.6088 (2)	0.0727 (7)	
H13	0.7827	0.5627	0.5692	0.087*	
C14	0.8284 (3)	0.2270 (2)	0.3940 (3)	0.0960 (9)	
H14A	0.8253	0.1882	0.3285	0.115*	0.58
H14B	0.8023	0.2933	0.3688	0.115*	0.58
H14C	0.8351	0.2980	0.3990	0.115*	0.42
H14D	0.8145	0.2068	0.3176	0.115*	0.42
C15	0.9688 (6)	0.2310 (5)	0.4575 (7)	0.118 (2)	0.58
H15A	0.9764	0.2644	0.5272	0.141*	0.58
H15B	1.0223	0.2667	0.4168	0.141*	0.58
C16	1.0198 (10)	0.1280 (6)	0.4782 (7)	0.138 (3)	0.58
H16A	0.9695	0.0943	0.5219	0.207*	0.58
H16B	1.1146	0.1289	0.5172	0.207*	0.58
H16C	1.0081	0.0946	0.4088	0.207*	0.58
C15'	0.9540 (9)	0.1753 (13)	0.4727 (13)	0.161 (6)	0.42
H15C	0.9605	0.1952	0.5483	0.194*	0.42
H15D	0.9395	0.1049	0.4682	0.194*	0.42
C16'	1.0849 (9)	0.1976 (10)	0.4471 (10)	0.149 (4)	0.42
H16D	1.1045	0.1473	0.3999	0.223*	0.42
H16E	1.1566	0.1999	0.5147	0.223*	0.42
H16F	1.0784	0.2599	0.4103	0.223*	0.42
C17	0.5680 (9)	0.0856 (5)	0.1674 (7)	0.148 (3)	0.66
H17A	0.5009	0.1024	0.2055	0.223*	0.66
H17B	0.5254	0.0807	0.0894	0.223*	0.66
H17C	0.6368	0.1356	0.1803	0.223*	0.66
C18	0.6292 (9)	−0.0070 (5)	0.2079 (5)	0.118 (2)	0.66
H18A	0.5617	−0.0588	0.1878	0.142*	0.66
H18B	0.7020	−0.0216	0.1737	0.142*	0.66
C17'	0.6268 (14)	0.0358 (14)	0.1313 (9)	0.137 (4)	0.34
H17D	0.7050	0.0778	0.1528	0.205*	0.34
H17E	0.5587	0.0665	0.0728	0.205*	0.34
H17F	0.6527	−0.0258	0.1058	0.205*	0.34
C18'	0.5711 (10)	0.0189 (12)	0.2276 (7)	0.099 (3)	0.34
H18C	0.5239	0.0772	0.2420	0.118*	0.34
H18D	0.5064	−0.0349	0.2119	0.118*	0.34
N1	0.56533 (17)	0.19870 (11)	0.54545 (13)	0.0526 (4)	
N2	0.70225 (18)	0.33593 (12)	0.52685 (15)	0.0586 (5)	
N3	0.7232 (2)	0.18964 (13)	0.44097 (17)	0.0680 (5)	
H3A	0.6966	0.1299	0.4271	0.082*	
O1	0.40323 (19)	0.20284 (12)	0.64207 (15)	0.0807 (5)	
O2	0.47273 (17)	0.41186 (11)	0.68384 (13)	0.0729 (5)	
O3	0.6821 (2)	−0.00452 (14)	0.32441 (15)	0.0915 (6)	
H3B	0.6693	−0.0577	0.3506	0.137*	

### Atomic displacement parameters (Å^2^)

**Table:**

	*U*^11^	*U*^22^	*U*^33^	*U*^12^	*U*^13^	*U*^23^
C1	0.0582 (12)	0.0497 (12)	0.0530 (11)	−0.0030 (9)	−0.0011 (9)	−0.0018 (9)
C2	0.0579 (12)	0.0437 (11)	0.0488 (10)	−0.0026 (9)	0.0118 (9)	−0.0019 (8)
C3	0.0608 (12)	0.0466 (12)	0.0537 (11)	0.0037 (9)	0.0028 (9)	0.0055 (9)
C4	0.0551 (12)	0.0461 (11)	0.0563 (11)	−0.0006 (9)	0.0076 (9)	0.0007 (9)
C5	0.0603 (12)	0.0448 (11)	0.0500 (10)	−0.0001 (9)	−0.0006 (9)	0.0004 (9)
C6	0.0698 (13)	0.0472 (12)	0.0582 (12)	0.0014 (10)	0.0115 (10)	−0.0089 (9)
C7	0.0677 (13)	0.0522 (12)	0.0563 (12)	−0.0050 (11)	0.0160 (10)	−0.0041 (10)
C8	0.0683 (13)	0.0435 (11)	0.0517 (11)	0.0035 (10)	−0.0061 (10)	−0.0004 (9)
C9	0.0775 (15)	0.0475 (13)	0.0591 (12)	0.0056 (11)	−0.0028 (11)	−0.0069 (10)
C10	0.0969 (19)	0.0583 (15)	0.0710 (15)	0.0152 (13)	0.0012 (13)	−0.0132 (12)
C11	0.115 (2)	0.0478 (14)	0.0770 (17)	0.0193 (15)	−0.0122 (16)	−0.0084 (12)
C12	0.111 (2)	0.0410 (13)	0.0850 (17)	0.0000 (13)	−0.0106 (16)	0.0040 (12)
C13	0.0867 (17)	0.0468 (13)	0.0709 (14)	−0.0030 (11)	−0.0030 (12)	0.0050 (10)
C14	0.094 (2)	0.0741 (18)	0.136 (3)	−0.0151 (16)	0.059 (2)	−0.0216 (17)
C15	0.082 (4)	0.139 (6)	0.149 (5)	−0.045 (4)	0.060 (4)	−0.051 (5)
C16	0.142 (6)	0.163 (7)	0.107 (4)	0.039 (5)	0.029 (4)	0.018 (4)
C15'	0.142 (9)	0.174 (10)	0.181 (9)	−0.029 (8)	0.065 (8)	0.033 (8)
C16'	0.098 (6)	0.193 (9)	0.156 (7)	0.000 (6)	0.037 (5)	0.007 (7)
C17	0.183 (6)	0.123 (5)	0.118 (4)	0.041 (4)	0.001 (4)	0.004 (4)
C18	0.155 (6)	0.110 (4)	0.094 (4)	0.021 (4)	0.042 (4)	−0.005 (3)
C17'	0.151 (8)	0.157 (9)	0.107 (7)	0.029 (7)	0.042 (6)	0.029 (7)
C18'	0.095 (6)	0.121 (8)	0.083 (6)	0.002 (6)	0.030 (5)	0.000 (6)
N1	0.0602 (10)	0.0416 (9)	0.0541 (9)	−0.0036 (7)	0.0120 (8)	−0.0030 (7)
N2	0.0621 (10)	0.0441 (10)	0.0661 (11)	−0.0044 (8)	0.0110 (8)	−0.0015 (8)
N3	0.0744 (12)	0.0507 (10)	0.0860 (13)	−0.0094 (9)	0.0339 (10)	−0.0097 (9)
O1	0.0978 (13)	0.0648 (11)	0.0927 (12)	−0.0184 (9)	0.0484 (11)	−0.0170 (9)
O2	0.0882 (11)	0.0563 (10)	0.0750 (10)	−0.0013 (8)	0.0233 (9)	−0.0165 (8)
O3	0.1105 (15)	0.0799 (12)	0.0767 (12)	−0.0173 (10)	0.0123 (11)	0.0047 (9)

### Geometric parameters (Å, º)

**Table:**

C1—C3^i^	1.373 (3)	C14—H14B	0.9700
C1—C2	1.376 (3)	C14—H14C	0.9700
C1—H1	0.9300	C14—H14D	0.9700
C2—C3	1.376 (3)	C15—C16	1.494 (8)
C2—N1	1.444 (2)	C15—H15A	0.9700
C3—C1^i^	1.373 (3)	C15—H15B	0.9700
C3—H3	0.9300	C16—H16A	0.9600
C4—N2	1.314 (3)	C16—H16B	0.9600
C4—N3	1.340 (3)	C16—H16C	0.9600
C4—N1	1.396 (3)	C15'—C16'	1.484 (9)
C5—N2	1.356 (3)	C15'—H15C	0.9700
C5—C6	1.359 (3)	C15'—H15D	0.9700
C5—C8	1.451 (3)	C16'—H16D	0.9600
C6—O2	1.380 (2)	C16'—H16E	0.9600
C6—C7	1.406 (3)	C16'—H16F	0.9600
C7—O1	1.221 (3)	C17—C18	1.439 (7)
C7—N1	1.412 (3)	C17—H17A	0.9600
C8—C9	1.387 (4)	C17—H17B	0.9600
C8—C13	1.391 (3)	C17—H17C	0.9600
C9—O2	1.378 (3)	C18—O3	1.415 (6)
C9—C10	1.379 (3)	C18—H18A	0.9700
C10—C11	1.372 (4)	C18—H18B	0.9700
C10—H10	0.9300	C17'—C18'	1.482 (9)
C11—C12	1.386 (4)	C17'—H17D	0.9600
C11—H11	0.9300	C17'—H17E	0.9600
C12—C13	1.375 (4)	C17'—H17F	0.9600
C12—H12	0.9300	C18'—O3	1.457 (8)
C13—H13	0.9300	C18'—H18C	0.9700
C14—C15	1.442 (6)	C18'—H18D	0.9700
C14—N3	1.446 (3)	N3—H3A	0.8600
C14—C15'	1.561 (9)	O3—H3B	0.8200
C14—H14A	0.9700		
			
C3^i^—C1—C2	119.50 (18)	N3—C14—H14D	111.9
C3^i^—C1—H1	120.3	C15'—C14—H14D	112.0
C2—C1—H1	120.3	H14A—C14—H14D	17.5
C3—C2—C1	120.85 (18)	H14B—C14—H14D	89.3
C3—C2—N1	119.49 (17)	H14C—C14—H14D	109.6
C1—C2—N1	119.65 (17)	C14—C15—C16	108.0 (6)
C1^i^—C3—C2	119.65 (18)	C14—C15—H15A	110.1
C1^i^—C3—H3	120.2	C16—C15—H15A	110.1
C2—C3—H3	120.2	C14—C15—H15B	110.1
N2—C4—N3	120.22 (19)	C16—C15—H15B	110.1
N2—C4—N1	122.93 (19)	H15A—C15—H15B	108.4
N3—C4—N1	116.85 (18)	C16'—C15'—C14	113.9 (9)
N2—C5—C6	125.20 (19)	C16'—C15'—H15C	108.8
N2—C5—C8	129.4 (2)	C14—C15'—H15C	108.8
C6—C5—C8	105.39 (19)	C16'—C15'—H15D	108.8
C5—C6—O2	113.20 (18)	C14—C15'—H15D	108.8
C5—C6—C7	122.9 (2)	H15C—C15'—H15D	107.7
O2—C6—C7	123.9 (2)	C15'—C16'—H16D	109.5
O1—C7—C6	128.7 (2)	C15'—C16'—H16E	109.5
O1—C7—N1	121.00 (19)	H16D—C16'—H16E	109.5
C6—C7—N1	110.25 (19)	C15'—C16'—H16F	109.5
C9—C8—C13	119.0 (2)	H16D—C16'—H16F	109.5
C9—C8—C5	105.22 (19)	H16E—C16'—H16F	109.5
C13—C8—C5	135.7 (2)	O3—C18—C17	110.6 (5)
O2—C9—C10	124.5 (3)	O3—C18—H18A	109.5
O2—C9—C8	111.97 (18)	C17—C18—H18A	109.5
C10—C9—C8	123.5 (2)	O3—C18—H18B	109.5
C11—C10—C9	116.3 (3)	C17—C18—H18B	109.5
C11—C10—H10	121.8	H18A—C18—H18B	108.1
C9—C10—H10	121.8	C18'—C17'—H17D	109.5
C10—C11—C12	121.5 (2)	C18'—C17'—H17E	109.5
C10—C11—H11	119.3	H17D—C17'—H17E	109.5
C12—C11—H11	119.3	C18'—C17'—H17F	109.5
C13—C12—C11	121.7 (3)	H17D—C17'—H17F	109.5
C13—C12—H12	119.2	H17E—C17'—H17F	109.5
C11—C12—H12	119.2	O3—C18'—C17'	109.3 (9)
C12—C13—C8	117.9 (3)	O3—C18'—H18C	109.8
C12—C13—H13	121.0	C17'—C18'—H18C	109.8
C8—C13—H13	121.0	O3—C18'—H18D	109.8
C15—C14—N3	121.6 (4)	C17'—C18'—H18D	109.8
C15—C14—C15'	30.8 (5)	H18C—C18'—H18D	108.3
N3—C14—C15'	99.0 (5)	C4—N1—C7	124.16 (18)
C15—C14—H14A	106.9	C4—N1—C2	120.16 (16)
N3—C14—H14A	106.9	C7—N1—C2	115.68 (16)
C15'—C14—H14A	97.2	C4—N2—C5	114.54 (18)
C15—C14—H14B	106.9	C4—N3—C14	122.8 (2)
N3—C14—H14B	106.9	C4—N3—H3A	118.6
C15'—C14—H14B	137.2	C14—N3—H3A	118.6
H14A—C14—H14B	106.7	C9—O2—C6	104.22 (18)
C15—C14—H14C	83.4	C18—O3—C18'	31.7 (4)
N3—C14—H14C	111.9	C18—O3—H3A	124.5
C15'—C14—H14C	112.2	C18'—O3—H3A	102.6
H14A—C14—H14C	125.7	C18—O3—H3B	109.5
H14B—C14—H14C	26.1	C18'—O3—H3B	110.8
C15—C14—H14D	114.7	H3A—O3—H3B	119.4
			
C3^i^—C1—C2—C3	0.2 (3)	N3—C14—C15'—C16'	179.6 (12)
C3^i^—C1—C2—N1	178.92 (18)	N2—C4—N1—C7	2.6 (3)
C1—C2—C3—C1^i^	−0.2 (4)	N3—C4—N1—C7	−176.89 (18)
N1—C2—C3—C1^i^	−178.92 (18)	N2—C4—N1—C2	−177.97 (18)
N2—C5—C6—O2	−178.42 (17)	N3—C4—N1—C2	2.6 (3)
C8—C5—C6—O2	0.5 (2)	O1—C7—N1—C4	179.69 (19)
N2—C5—C6—C7	0.2 (3)	C6—C7—N1—C4	−0.5 (3)
C8—C5—C6—C7	179.07 (19)	O1—C7—N1—C2	0.2 (3)
C5—C6—C7—O1	179.0 (2)	C6—C7—N1—C2	180.00 (17)
O2—C6—C7—O1	−2.5 (4)	C3—C2—N1—C4	−101.3 (2)
C5—C6—C7—N1	−0.7 (3)	C1—C2—N1—C4	80.0 (2)
O2—C6—C7—N1	177.70 (18)	C3—C2—N1—C7	78.2 (2)
N2—C5—C8—C9	178.42 (19)	C1—C2—N1—C7	−100.5 (2)
C6—C5—C8—C9	−0.4 (2)	N3—C4—N2—C5	176.47 (18)
N2—C5—C8—C13	−0.4 (4)	N1—C4—N2—C5	−3.0 (3)
C6—C5—C8—C13	−179.3 (2)	C6—C5—N2—C4	1.7 (3)
C13—C8—C9—O2	179.31 (18)	C8—C5—N2—C4	−176.91 (18)
C5—C8—C9—O2	0.2 (2)	N2—C4—N3—C14	0.8 (3)
C13—C8—C9—C10	−1.3 (3)	N1—C4—N3—C14	−179.7 (2)
C5—C8—C9—C10	179.6 (2)	C15—C14—N3—C4	81.6 (4)
O2—C9—C10—C11	179.4 (2)	C15'—C14—N3—C4	104.2 (8)
C8—C9—C10—C11	0.1 (3)	C10—C9—O2—C6	−179.3 (2)
C9—C10—C11—C12	0.7 (4)	C8—C9—O2—C6	0.0 (2)
C10—C11—C12—C13	−0.2 (4)	C5—C6—O2—C9	−0.3 (2)
C11—C12—C13—C8	−0.9 (3)	C7—C6—O2—C9	−178.9 (2)
C9—C8—C13—C12	1.6 (3)	C17—C18—O3—C18'	44.0 (11)
C5—C8—C13—C12	−179.6 (2)	C17—C18—O3—H3A	−8.6
N3—C14—C15—C16	68.1 (6)	C17'—C18'—O3—C18	−29.5 (10)
C15'—C14—C15—C16	20.3 (14)	C17'—C18'—O3—H3A	108.4
C15—C14—C15'—C16'	−40.1 (9)		

Symmetry code: (i) −*x*+1, −*y*, −*z*+1.

### Hydrogen-bond geometry (Å, º)

**Table:**

*D*—H···*A*	*D*—H	H···*A*	*D*···*A*	*D*—H···*A*
N3—H3a···O3	0.86	2.22	2.996 (3)	150
O3—H3b···O1^i^	0.82	2.12	2.903 (3)	159

Symmetry code: (i) −*x*+1, −*y*, −*z*+1.
